# Two Photon–Pumped Whispering‐Gallery Mode Lasing and Dynamic Regulation

**DOI:** 10.1002/advs.201900916

**Published:** 2019-09-30

**Authors:** Junfeng Lu, Fangtao Li, Wenda Ma, Jufang Hu, Yiyao Peng, Zheng Yang, Qiushuo Chen, Chunxiang Xu, Caofeng Pan, Zhong Lin Wang

**Affiliations:** ^1^ CAS Center for Excellence in Nanoscience Beijing Key Laboratory of Micro‐Nano Energy and Sensor Beijing Institute of Nanoenergy and Nanosystems Chinese Academy of Sciences Beijing 100083 P. R. China; ^2^ School of Nanoscience and Technology University of Chinese Academy of Sciences Beijing 100049 P. R. China; ^3^ State Key Laboratory of Bioelectronics School of Biological Science and Medical Engineering Southeast University Nanjing 210096 P. R. China; ^4^ School of Materials Science and Engineering Georgia Institute of Technology Atlanta GA 30332‐0245 USA

**Keywords:** frequency upconversion lasers, mode regulation, nonlinear optics, piezoelectric polarization effect, two‐photon absorption, whispering‐gallery mode

## Abstract

Realizing the dynamic regulation of nonlinear optical signals has a great scientific significance for the development of new‐type nonlinear optoelectronic devices and expands its application in the field of laser technology, spectroscopy, material structure analysis, etc. Here, two photon absorption–induced whispering‐gallery mode lasing from a single ZnO microresonator with a relatively low lasing threshold (15 µW) and high quality factor (*Q* ≈ 3200) under ambient conditions is demonstrated. Furthermore, success is achieved in obtaining the dynamic regulation of two photon–pumped lasing mode in the UV gain region. The corresponding resonant wavelength can be tuned dynamically from 388.99 and 391.12 to 390.01 and 392.12 nm for TE_33_ and TE_32_ modes, respectively. This work provides a new strategy for building high‐performance mode‐adjustable frequency upconversion lasers.

## Graphical abstract

**A high quality (*Q* ≈ 3200), low threshold (*P*_th_ ≈ 15 µW) frequency upconverting lasing output** is achieved pumped by two‐photon absorption. Also, the resonant wavelength of lasing mode can be regulated dynamically to succeed through the piezoelectric polarization effect induced by the external mechanical disturbance. It provides a new strategy for building high‐performance mode‐adjustable frequency upconversion lasers.

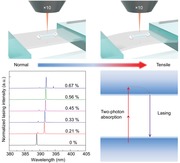

As an important branch of modern optics, nonlinear optics has a wide range of applications in laser technology, optical communication, and integrated optics. From the perspective of its development, the rise of nonlinear optics is inseparable from the emergence of lasers, and the two complement each other. In 1960, Maiman^1^ successfully built the first ruby laser at a wavelength of 694.3 nm, which greatly promoted the development from science to technology. On this basis, Franken et al. focused the ruby laser beam of 694.3 nm on a crystal‐line quartz and observed the second harmonic generation (SHG) at 347.2 nm for the first time in 1961.^2^ Since then, amount of nonlinear optical phenomena such as optical harmonics, optical sum and difference frequencies, optical parametric amplification and oscillation, multiphoton absorption (MPA), etc., have been discovered in succession,^3^, ^4^, ^5^, ^6^, ^7^ which is closely related to the development of laser tuning technology and ultrashort pulse laser technology.

As a typical representative of third‐generation semiconductor materials, ZnO has an exciton binding energy of up to 60 meV and a wide direct bandgap of 3.37 eV, making it an excellent candidate for building short‐wavelength laser devices.^8^, ^9^, ^10^, ^11^, ^12^ In addition, the high optical damage threshold of ZnO makes it possible to investigate the nonlinear optical phenomenon under extremely strong excitation conditions with short pulsed intense lasers. In the past two decades, a large number of second‐order and third‐order nonlinear optical phenomena on non‐centrosymmetric wurtzite structural ZnO, including SHG, third harmonic generation, TPA, MPA, and multiphoton emission have been reported.^13^, ^14^, ^15^, ^16^, ^17^ In particular, the utilizing of two‐photon absorption process as the excitation route to achieve stimulated emission is considered as a promising strategy for obtaining frequency upconversion lasers.^18^, ^19^, ^20^, ^21^ However, the dynamic regulation of nonlinear optical signals has great potential applications in constructing new‐type nonlinear optoelectronic devices such as frequency upconversion lasers, nonlinear optical switches, optical parametric amplifier, optical sensors, etc., which has become the focus of researchers. In recent years, the researches on piezotronics and piezophototronics based on the piezoelectric effect of non‐centrosymmetric wurtzite structural ZnO have been widely reported by Wang and Song.^22^, ^23^, ^24^, ^25^, ^26^ The piezopotential can be used to effectively regulate the interface band structure and control the charge separation, transport, and recombination of an optoelectronic processes for achieving superior performance.^27^, ^28^, ^29^, ^30^, ^31^, ^32^, ^33^ Moreover, the change of the internal dipole moment of the ZnO microcrystal generated by the external mechanical disturbance has a direct influence on the refractive index (dielectric constant) of the medium, which is expected to be used for the dynamic regulation of the lasing mode.^34^, ^35^

Here, we have developed a new strategy for dynamically regulating two photon–pumped whispering‐gallery mode (WGM) lasing by piezoelectric polarization effect based on single ZnO microresonator. The obtained high‐quality (*Q* ≈ 3200) two photon–pumped ZnO lasing mode of TE_33_ and TE_32_ can be dynamically tuned from 388.99 and 391.12 to 390.01 and 392.12 nm as the tensile strain varies from 0% to 0.67%. The measurement of polarized angle–resolved and strain‐loaded Raman spectra has been performed to verify the geometric anisotropy of ZnO microcrystals, which provides a direct evidence for the deviation of the positive and negative charge centers in unit cell with an asymmetric central structure, and the generation of the dipole moment under external mechanical perturbations. Meanwhile, according to the plane wave model, we systematically analyzed the oscillation of the light wave, the selection of resonant mode, and the reason of mode shift in ZnO microcavity, not only providing a scientific basis for constructing a class of mode‐adjustable frequency upconversion laser, but also verifying that the method of mode regulation based on piezoelectric polarization effect is universal.

**Figure** **1**a shows the typical transmission electron microscopy (TEM) image of the cross‐sectional ZnO microdisk prepared by focused ion beam (FIB) milling equipped on the dual‐beam electron microscope (FEI, Helios NanoLab 600i). First, in order to reduce the damage of the ion beam to the sample and facilitate the preparation of TEM samples, the metal platinum (Pt) is filled and wrapped around the selected ZnO microrod by electron beam deposition. Then, the selected area is separated from the sample body by focused gallium ion beam with a large beam current, and transferred and fixed onto the Cu grid. Finally, a small current‐focused gallium ion beam is used to thin the transferred sample to several hundred nanometers. The cross‐section of the as‐prepared sample exhibits a distinct hexagonal structure with an internal angle of 120°, which also can be observed from the 45° tilted scanning electron microscopy (SEM) image insetted in Figure 1a. Figure 1b shows the Zn and O element mapping collected from the yellow rectangle region marked in Figure 1a. The distinct boundary and uniform distribution of Zn element mapping is corresponding to the profile of the as‐prepared sample. The uneven distribution of O element measured here is mainly caused by two factors. The first one is the small relative atomic mass of the oxygen atom. The second one is due to uneven cutting on the left and right sides of the ZnO microdisk during the thinning process. To further demonstrate the structural characteristics of ZnO microrod, high‐resolution TEM (HRTEM) image and the corresponding selected area electron diffraction (SAED) pattern of the as‐prepared ZnO microdisk are measured, as shown in Figure 1d,e. Combined with the axial structural characterization (see Figure S1 in the Supporting Information), it can be observed that single ZnO microrod preferentially grows along the [0001] direction, and exhibits a hexagonal symmetry structure as the axis. Figure 1c shows the typical X‐ray diffraction (XRD) pattern of ZnO microrod arrays. All the diffraction peaks are consistent with the indexes of the wurtzite‐phased ZnO (JCPDS no. 36‐1451) shown in the inset of Figure 1c. And, the narrow full‐width at half‐maximum (FWHM) of diffraction peaks implies high crystallinity of ZnO microrods synthesized at a high temperature, which is an ideal carrier for optical microcavities. In addition, ZnO is also a gain medium with excellent optical properties. The UV–vis absorption and photoluminescence (PL) spectra at short wavelength region are presented in Figure 1f. A strong near‐band edge (NBE) emission centered at ≈390 nm can be observed, which will provide sufficient optical gain for light amplification confined in the microcavity.

**Figure 1:**
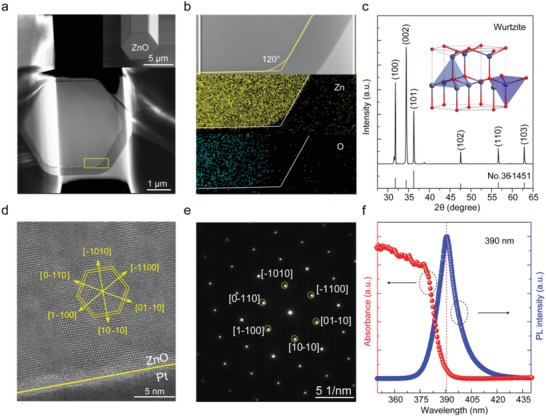
Structural characteristics and optical properties of the as‐prepared ZnO microrod. a,d,e) TEM, HRTEM, and SAED of the cross‐sectional ZnO microdisk prepared by FIB method, inset: SEM image of the cross‐sectional ZnO microrod. b,c,f) Elemental mapping, XRD, absorption and PL spectrum of ZnO, inset: schematic diagram of the wurtzite‐structural ZnO.

To demonstrate the geometric anisotropy of ZnO microcrystals, Raman characteristics under different polarized light angles and external tensile strains are measured at room temperature. **Figure** **2**a shows schematic diagram of two typical Raman characteristic peaks of A_1TO_ and E_2H_ phonon modes for wurtzite‐structural ZnO single crystals. The vibration direction of the former is parallel the [0001] direction, while that of the latter is perpendicular to the [0001] direction. The polarization angle of the incident light can be regulated by rotating the angle of the half wave plate placed at the front confocal micro‐Raman microscopy system, as shown in Figure 2b. Theta (θ) represents the included angle between the polarized light and the *c*‐axis of single ZnO microrod. Figure 2c shows 2D color plots of polarized angle–dependent Raman spectra under the strain‐free state insetted with the polar plot of Raman intensity for E_2L_, E_2H_ dependence on the polarization angle of incident light by rotating the λ/2 wave plate. The measured polarized light angle–dependent Raman intensity of E_2L_, E_2H_ exhibits extremely strong polarization characteristics, further verifying that wurtzite‐structural ZnO is a kind of polar crystal with asymmetry. As the polarization (E_∥_) configuration is parallel to *c* axis of ZnO (θ = 0°), a predominant phonon mode of A_1TO_ at 374.0 cm^−1^ can be observed. When θ = 90° (E_⊥_), the modes of E_2L_ and E_2H_ with the phonon frequencies of 95.0 and 434.6 cm^−1^ will dominate (see Figure S2 in the Supporting Information). The similar polarization‐dependent Raman scattering signals of single strain–loaded ZnO microrod has been obtained shown in Figure 2d, indicating that the applied external stress did not change the intrinsic crystal structure of ZnO, but shifted the phonon resonant frequency of E_2L_ and E_2H_ modes. Figure 2e shows the evolution of Raman spectra with increasing tensile strain from 0% to 0.50%. It is worth noting that not all the photon modes will shift with increasing of the tensile strain due to the otherness of the stain loading method in different previous reports.^36^, ^37^ Figure 2f shows the dependence of photon frequency shifts of E_2L_, E_2H_, and A_1TO_ on the tensile strain. Apart from A_1TO_, the photon frequency of E_2L_ and E_2H_ modes linearly shift toward the low wavenumber as the tensile strain increases. To discuss the frequency behaviors of A_1TO_ phonon in ZnO microrod, a model of harmonic oscillator in a 1D system can be used for analysis. According to the classical atomic model, the law of electron motion in an atom can be described by an electric dipole of simple harmonic oscillation, namely harmonic oscillator. In the 1D case, the harmonic potential energy can be expressed as *U*(*x*) = *Kx* ^2^/2, where *K* is the elastic coefficient and *x* is the distance from the equilibrium position. The corresponding elastic restoring force can be expressed as *F*(*x*) = − ∂*U*(*x*)/∂*x*  = −*Kx*, and then the dynamic equation of oscillator can be inferred as *m*(d^2^ *x*/d*t* ^2^) = −*Kx*, where *m* is a mass of oscillator. When a small stretching force *F*_0_ is applied, it will cause a displacement *x*_0_ away from the equilibrium position with *F*_0_  =   −*Kx*_0_, then the dynamic equation should be rewritten as *m*(d^2^ *x*/d*t* ^2^) = −*Kx* + *F*_0_, which can be reformed as [d^2^(*x* + *x*_0_)]/d*t* ^2^  + (*K*/*m*)(*x* + *x*_0_) = 0. The intrinsic vibration frequency of  ω_0_ = (*K*/*m*)^1/2^ cannot be changed with external force or deformation along the vibration direction, which is consistent with the results observed in our experiment. Therefore, the measurement of Raman scattering signals reveals that the uniaxial tensile strain along the *c*‐axis direction will result in the relative positional deviation of the positive and negative ions in the ZnO lattice, and further affecting the phonon frequency vibrating perpendicular to the *c*‐axis, which provides a direct evidence for the deviation of the positive and negative charge centers in unit cell with an asymmetric central structure, and the generation of the dipole moment under external mechanical perturbations.

**Figure 2:**
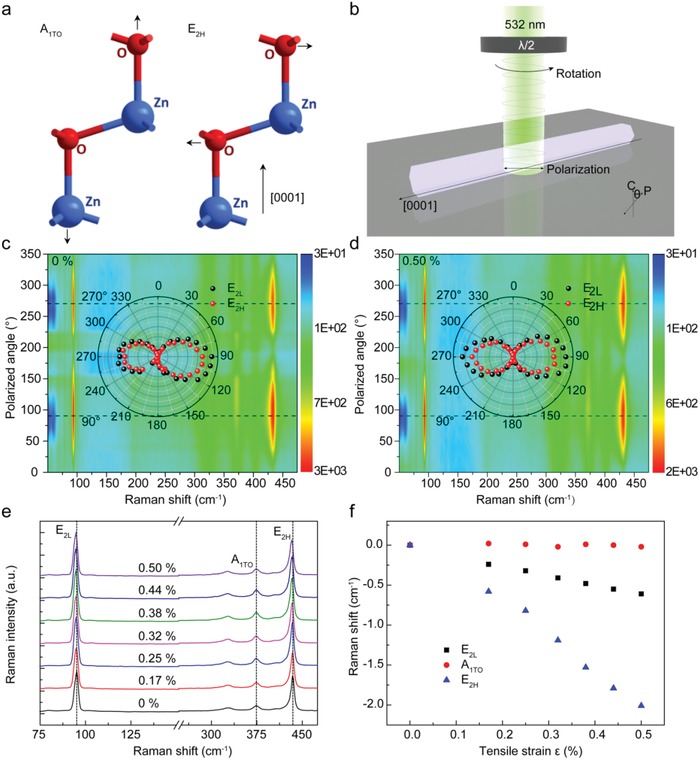
Raman characteristics of single ZnO microrod. Schematic diagram of a) atomic vibrations for A_1TO_ and E_2H_ phonon modes and b) Raman measurement on wurtzite ZnO. 2D color plots of polarized angle–dependent Raman spectra at the cases of c) normal and d) tensile states by rotating the λ/2 wave plate. Inset: polar plot of Raman intensity for E_2L_, E_2H_ with rotating the λ/2 wave plate at normal and tensile cases. e) Raman peaks evolution under tensile strain from 0% to 0.50%. f) Phonon frequency shifts of E_2L_, E_2H_, and A_1TO_ as a function of tensile strain value.

In order to demonstrate the nonlinear optical characteristics of monocrystalline ZnO microcavity, the two photon–pumped lasing experiment have been carried out by using a 710 nm femtosecond laser as an excitation source, as shown in the schematic diagram of **Figure** **3**a. Figure 3b shows the 2D color plots of lasing spectra as a function of pumping power for a single ZnO microrod with a diameter of ≈4.5 µm. At low pumping power (*P* < 15 µW), a broad violet emission centered at ≈388 nm with a FWHM of ≈10 nm can be observed, which origins from NBE radiation transition of ZnO, namely spontaneous emission (SPE). As pumping power increases and exceeds 15 µW, some discrete sharp peaks (red line in Figure 3b) appear and grow rapidly, much higher than SPE (black line in Figure 3b). Figure 3c clearly demonstrates the evolution of ZnO emission spectra with increasing of pumping power. The inset in Figure 3c plots the light‐in‐light‐out (L–L) curve and FWHM data as a function of pumping power, indicating the threshold behavior. The value of lasing threshold can be estimated as *P*_th_ ≈ 15 µW. It can be found that the FWHM almost maintains a constant value of ≈10 nm below *P*_th_. When *P* ≥ *P*_th_, the emission intensity increases dramatically and the value of FWHM drops suddenly by two orders of magnitude, suggesting the occurrence of lasing process. At *P* = 15 µW, the FWHM of the dominant lasing mode is 0.12 nm obtained by Gaussian fitting. The lasing quality factor (*Q*) can be estimated as *Q* = λ/*δλ* ≈ 3200.

**Figure 3:**
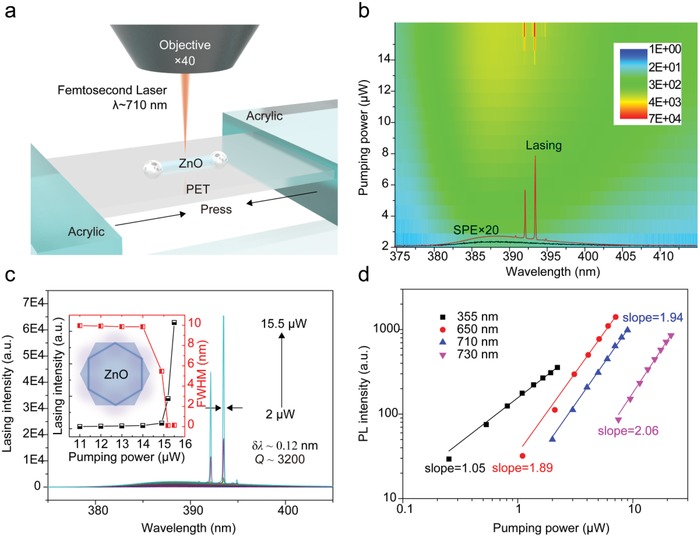
Two photon–pumped WGM lasing. a) Schematic of single ZnO microrod fixed on the flexible PET substrate at the case of normal state pumped by 710 nm laser excitation (≈190 fs, 1 kHz). b) 2D color plots of lasing spectra for single ZnO microrod as a function of pumping power at an excitation wavelength of 710 nm. c) Pump power–dependent lasing spectra from the ZnO microcavity, inset: dependence of the integrated lasing intensity and FWHM on pumping power, showing a threshold behavior. d) Pump‐power dependence of integrated PL intensity under different excitation wavelengths of 355, 650, 710, and 730 nm, respectively.

To further confirm the role of two‐photon absorption as an excitation source, PL spectra of the same sample excited by different wavelengths of 355, 650, 710, and 730 nm have been measured (see Figure S3 in the Supporting Information). Figure 3d plots the dependence of emission intensity under different excitation wavelengths on the pumping power. The relationship between the output intensity and input power can be described by a power exponential function of *I* ∝ *P* ^γ^, where the power‐law exponent γ is 1.05, 1.89, 1.94, and 2.06 for NBE emission excited by 355, 650, 710, and 730 nm, respectively. Obviously, for the excitation wavelength at 355 nm, the intensity of NBE emission and pumping power show a similar linear relationship, indicating that the energy of a single photon is sufficient for a single electron to complete the interband transition. And, for the excitation wavelength located at the red side of ZnO NBE emission, the PL intensity demonstrates the quadratic power dependence with the slopes of ≈2.0, further implying the participation of the two‐photon absorption process and to be the primary excitation pathway (see Figure S3e in the Supporting Information for the transition mechanism).

In order to achieve dynamic regulating of two photon–pumped ZnO WGM lasing, a home‐built acrylic carrier driven by a high‐precision manual stage is used to apple stress to single ZnO microrod fixed on the polyethylene terephthalate (PET) substrate along the *c*‐axis, as shown in **Figure** **4**a. In this case, a smaller ZnO microrod with a diameter of ≈2.2 µm is served as an optical resonant microcavity. The power‐dependent lasing spectra under the normal and tensile states have been measured through the same confocal microphotoluminescence system coupled with a femtosecond (fs) pulsed laser at 650 nm as the pumping source (see Figure S5a in the Supporting Information). Here, we utilize the plane wave model^38^, ^39^ to analyze the resonant mode and corresponding wavelength. The mode number can be expressed as $\;N\;=\;3\sqrt{3}nD\text{/}2\lambda \;-\;(6\text{/}\pi ){\text{tan}}^{-1}\left(n\sqrt{3n^{3}\;-\;4}\right)$ for hexagonal WGM cavity, where *D* is the diameter of the microresonator, λ is the resonant wavelength, and *n* is the refractive index for TE mode at the corresponding wavelength which can be described by Sellmeier's dispersion function of ZnO.^40^ The calculated mode number can be assigned as 33 and 32 for the resonant wavelength at 388.99 and 391.12 nm with the refractive index of 2.497 and 2.439 under the normal state, respectively (see Figure S4 in the Supporting Information).Figure 4b shows the evolution of lasing spectra with increasing of tensile strain from 0% to 0.67%. The obvious redshift of the resonant wavelength can be observed, which is consistent with the results of our previous reports pumped by single photon.^34^, ^35^ The resonant wavelengths of TE_33_ and TE_32_ modes shift from 388.99 and 391.12 to 390.01 and 392.12 nm in the long‐wave direction, respectively, as shown in Figure 4c. It can be seen from the mode calculated formula that only two facts of cavity size (*D*) and refractive index (*n*) have an effect on the wavelength of the resonant mode. If only considering the change in cavity size induced by Poisson effect of ZnO, the resonant wavelengths of TE_33_ and TE_32_ modes can be calculated and listed in Table S1 (Supporting Information). The reduced cavity size will lead to the blueshift of the resonant wavelength, contrary to our experimental observations. Therefore, the change of refractive index induced by the piezoelectric polarization effect is the dominant factor that causes the resonant wavelength to move. To further verify our inference, the refractive indexes at different tensile strains for TE_33_ and TE_32_ modes have been calculated, according to the formula mentioned before (see Figure S4 in the Supporting Information). The result shows a positive correlation between refractive index and the applied tensile strain. In addition, the measurement results of stress direction perpendicular to the *c*‐axis of ZnO further provide an efficient evidence for refractive index–induced mode regulation pumped by two photon (see Figure S7 in the Supporting Information). It is worth noting that the two photon–pumped ZnO WGM lasing has a stronger excitation power dependence than that pumped by single photon. The higher pump power is required to achieve stimulated emission at the case of strain application, which is more pronounced than single photon pumping. The generation of the piezoelectric polarization field has a certain influence on the exciton binding energy, which causes the positive and negative charges to separate, thus affecting the lasing threshold. But, the lasing quality is not affected by the piezoelectric polarization field and remains above 3000 (see Figure S5 in the Supporting Information). Figure 4d shows the dependence of PL intensity on pumping power at the tensile strain of 0% and 0.67%. With increasing of tensile strain, a slight increase of the power‐law exponent can be observed, but still maintains near‐quadratic increase, implying the important role of TPA in the excitation process, which also directly reflects the third‐order nonlinear optical characteristics of ZnO. It mainly manifests as the nonlinear refraction and the nonlinear absorption corresponding to the real and imaginary part of polarizability in ZnO, respectively. As mentioned before, the separation of positive and negative charge in the crystal caused by external perturbation will generate dipole moment, change the polarizability, and effectively regulate the refractive index and nonlinear absorption of the material, which provides a new effective strategy for dynamic regulation of nonlinear optical response.

**Figure 4:**
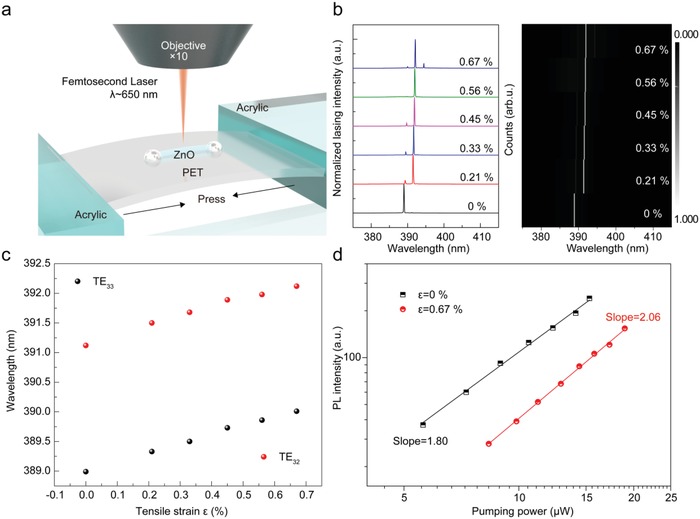
Dynamically regulated two photon–pumped WGM lasing. a) Schematic of single ZnO microrod fixed on the flexible PET substrate at the case of tensile state pumped by 650 nm laser excitation (≈190 fs, 1 kHz). b) Normalized lasing spectra (left panel) and mapping (right panel) for single ZnO microcavity under different tensile strains from 0% to 0.67%. c) The resonant wavelength of TE_33_ and TE_32_ modes as a function of tensile strain value. d) Integrated PL intensity of ZnO NBE emission under two different statements (ε = 0% and 0.67%) as a function of pumping power.

In summary, we obtained high‐quality two photon–pumped WGM lasing in ZnO microrod with a *Q* factor of ≈3200 under the excitation of 710 nm fs pulse laser. The measurement of polarized angle–dependent Raman spectra demonstrates that 1D ZnO microrod has a strong polarity along the *c*‐axis with an asymmetric central structure. As the tensile strain increases along the axial direction, the photon frequency of E_2L_ and E_2H_ modes linearly shift toward the low wavenumber, while A_1TO_ phonon remains stationary which has been analyzed by using the model of simple harmonic oscillated electric dipole. In addition, we realized the dynamic regulation of two photon–pumped lasing mode by piezoelectric polarization effect and clarified the regulation mechanism systematically, which provides an important scientific basis for the development of new nonlinear optoelectronic devices, such as high‐performance mode‐adjustable frequency upconversion lasers, nonlinear optical switches, optical parametric amplifier, and optical sensors, also expanding the application of piezoelectric polarization effect to the dynamic regulation of lasing mode in the field of nonlinear optics.

## Experimental Section

*Fabrication of ZnO Microrods*: The single‐crystal ZnO microrod arrays were synthesized by the vapor‐phase transport method, as reported in the previous works.^41^, ^42^ At a high reaction temperature of 1050 °C, a large amount of zinc vapor was reduced from the reaction source consisted of ZnO and graphite powder, and condensed on the Si substrate. When saturated, the ZnO seed crystals would precipitate out under the oxidation of oxygen molecules, and further absorbed Zn vapor, inducing the growth of ZnO microrods along the *c*‐axis. When the reaction was continued for 45 min, high‐quality ZnO microcavities could be obtained. Subsequently, a single ZnO microrod with a perfect hexagonal prism structure was transferred onto a flexible PET substrate and fixed at both ends with epoxy glue. The morphology and structure of ZnO microrods were characterized by SEM, TEM, and XRD.

*Optical Characterization*: The optically pumped lasing measurements were carried out on a highly integrated microsystem. A wavelength tunable fs laser (pulse length ≈ 190 fs, repetition rate ≈ 1 kHz) was employed as the excitation source and focused onto the samples through an upright microscope (Zeiss M1) equipped with a 10× (40×) objective. The two photon–pumped lasing emission excited by femtosecond pulses at 710 nm was collected by the same objective and analyzed using a charge‐coupled device (CCD) detector and an optical multichannel analyzer (Andor, SR‐500i‐D1‐R) with a 1200 g mm^−1^ grating. The Raman spectra of single ZnO microrod excited by a 532 nm continuous wave (CW) laser were measured by a confocal micro‐Raman microscopy system (HORIBA, LabRAM HR Evolution) with a spectral resolution of 0.65 cm^−1^. All the measurements were performed at room temperature.

*Mode Analysis*: In this case, the Sellmeier's dispersion function of ZnO was used to describe the relationship between refractive index and wavelength, $n(\lambda )\;=\;{\left(1\;+\;\frac{2.4885\lambda^{2}}{\lambda^{2}\;-\;102.30^{2}}\;+\;\frac{0.215\lambda^{2}}{\lambda^{2}\;-\;372.60^{2}}\;+\;\frac{0.2550\lambda^{2}}{\lambda^{2}\;-\;1850^{2}}\right)}^{1/2}$, meanwhile, combined with the plane wave model for hexagonal WGM cavity, the mode number *N* and the corresponding resonant wavelength λ could be deduced by the function of $N\;=\;3\sqrt{3}nD\text{/}2\lambda \;-\;(6\text{/}\pi ){\text{tan}}^{-1}\;\left(n\sqrt{3n^{3}\;-\;4}\right)$, where *D* is the diameter of cavity and *n* is the dispersion relation of ZnO.

## Conflict of Interest

The authors declare no conflict of interest.

## Supporting information

Supplementary
