# Supplementary material for: Two Photon–Pumped Whispering‐Gallery Mode Lasing and Dynamic Regulation
Source: Adv Sci (Weinh). 2019 Sep 30;6(22):1900916. doi: 10.1002/advs.201900916 (PMC6864518; doi:10.1002/advs.201900916)
Supplement: Supplementary file 1 — Supplementary [file ADVS-6-1900916-s001.pdf]

Copyright WILEY-VCH Verlag GmbH & Co. KGaA, 69469 Weinheim, Germany, 2019.

Supporting Information

for *Adv. Sci.,* DOI: 10.1002/advs.201900916

Two Photon–Pumped Whispering-Gallery Mode Lasing and Dynamic Regulation

Junfeng Lu, Fangtao Li, Wenda Ma, Jufang Hu, Yiyao Peng, Zheng Yang, Qiushuo Chen, Chunxiang Xu, Caofeng Pan, and Zhong Lin Wang**

Copyright WILEY-VCH Verlag GmbH & Co. KGaA, 69469 Weinheim, Germany, 2019.

Supporting Information

Two-photon-pumped whispering-gallery mode lasing and dynamic regulation

Junfeng Lu[†, ‡], Fangtao Li[†], Wenda Ma[†, ‡], Jufang Hu[†], Yiyao Peng[†, ‡], Zheng Yang[†, ‡], Qiushuo Chen[†], Chunxiang Xu[|], Caofeng Pan[†, ‡,], Zhong Lin Wang[†, ‡, ‖, *]*

Contents

A. **Structural characteristics of the sample prepared by FIB in *c*-axial.**

B. **Polarized-angle dependent Raman spectra under normal and tensile states.**

C. **Excitation wavelength-dependent PL spectra and transition mechanism.**

D. **Mode calculation in plane wave model.**

E. **Power-dependent lasing spectra under normal and tensile states.**

F. **Strain-induced refractive index variation and mode shift.**

G. **The calculated mode wavelength with decreasing of diameter.**

H. **Lasing spectra of stress direction perpendicular to *c*-axis of ZnO.**

I. **Power dependent lasing spectra and L-L curve at P⊥C and P∥C state.**

A. Structural characteristics of the sample prepared by FIB in *c*-axial.

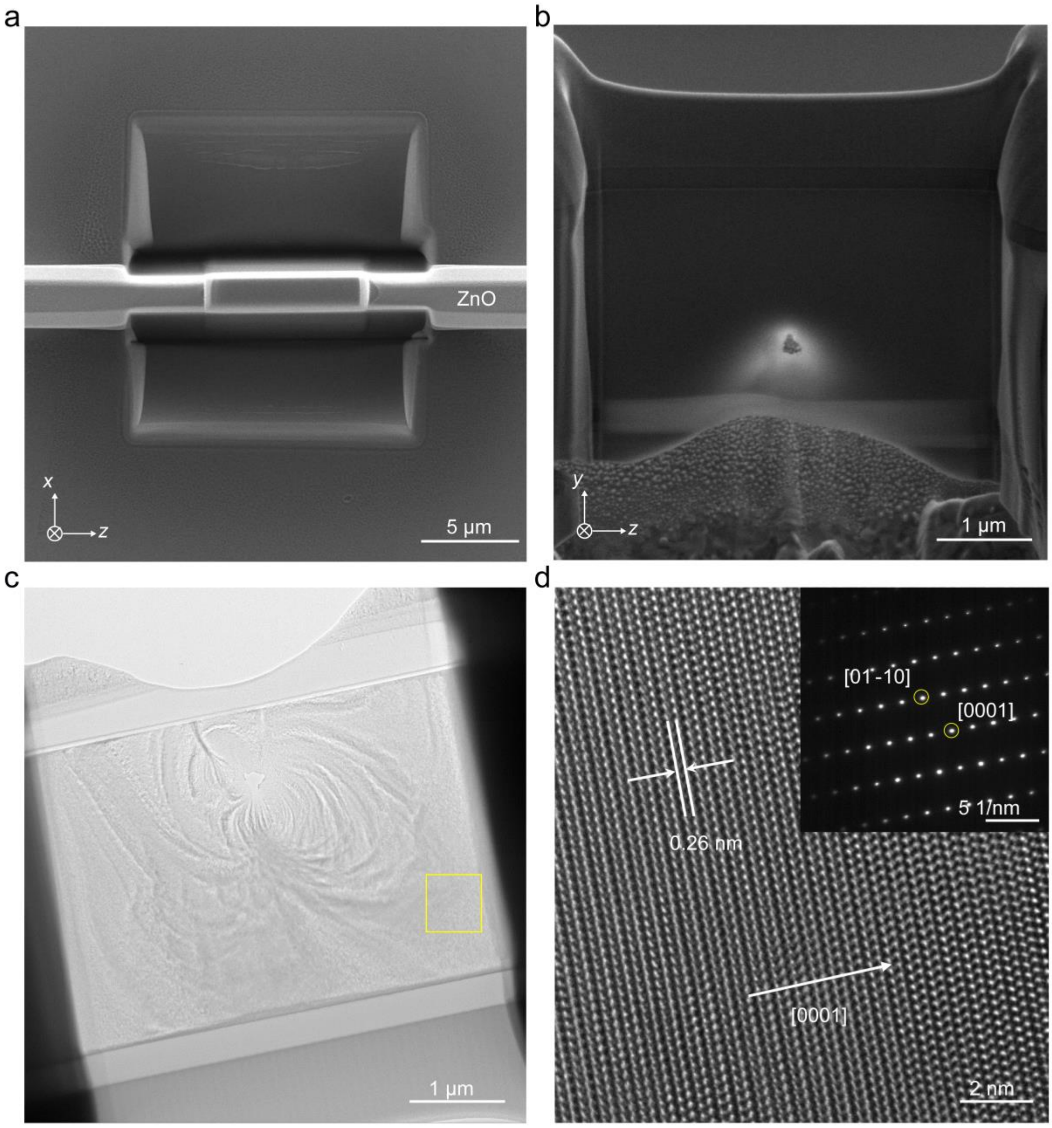

Figure S1. (a, b) SEM image of the ZnO microrod in *x-z* and *y-z plane*. (c, d) TEM and HRTEM image of the corresponding ZnO sample prepared by FIB method, inset: SEAD image of the same sample.

B. Polarized-angle dependent Raman spectra under normal and tensile states.

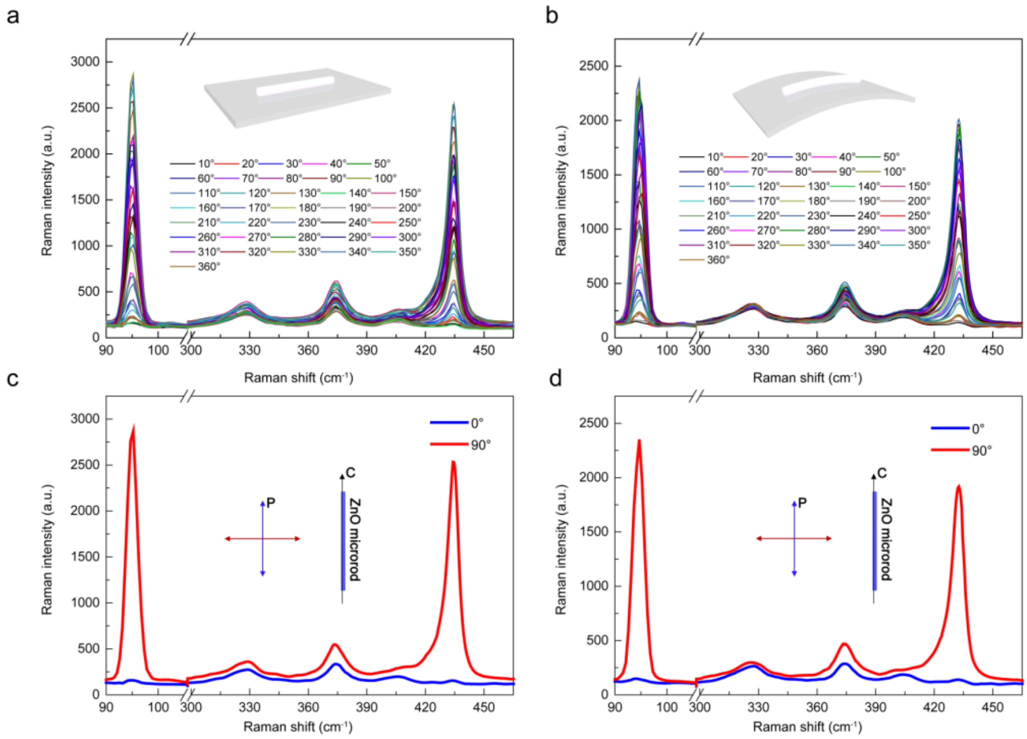

Figure S2. Polarized-angle dependent Raman spectra at the cases of normal (a) and tensile (b) states. Raman spectra of polarized direction parallel (blue line) and perpendicular (red line) to *C* axis of ZnO under normal (c) and tensile (d) states, respectively.

C. Excitation wavelength-dependent PL spectra and transition mechanism.

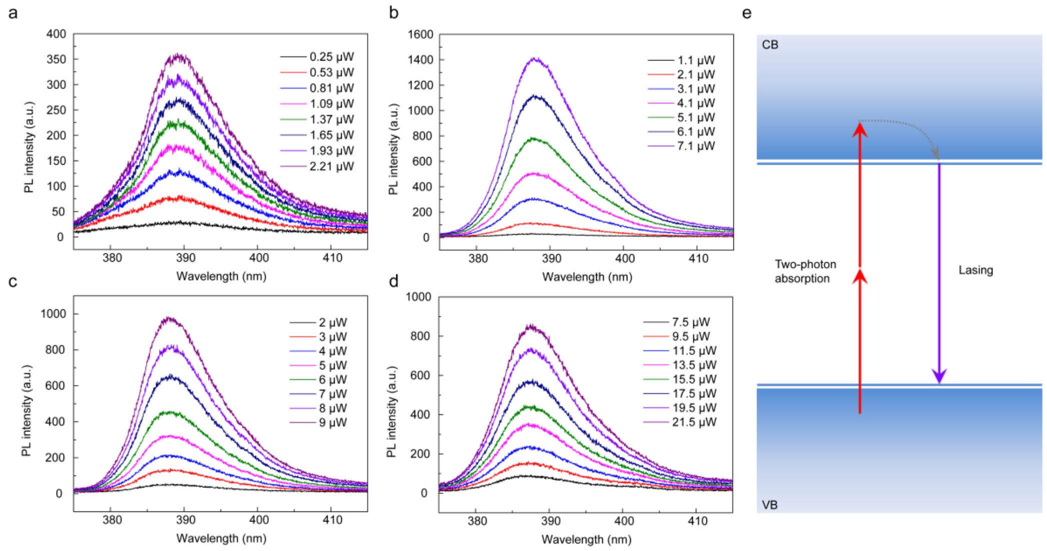

Figure S3. Power-dependent PL spectra of single ZnO microcavity with different excitation wavelengths of 355 (a), 650 (b), 710 (c) and 730 (d) nm. (e) Schematic diagram of two-photon-pumped ZnO stimulated emission.

D. Mode calculation in plane wave model.

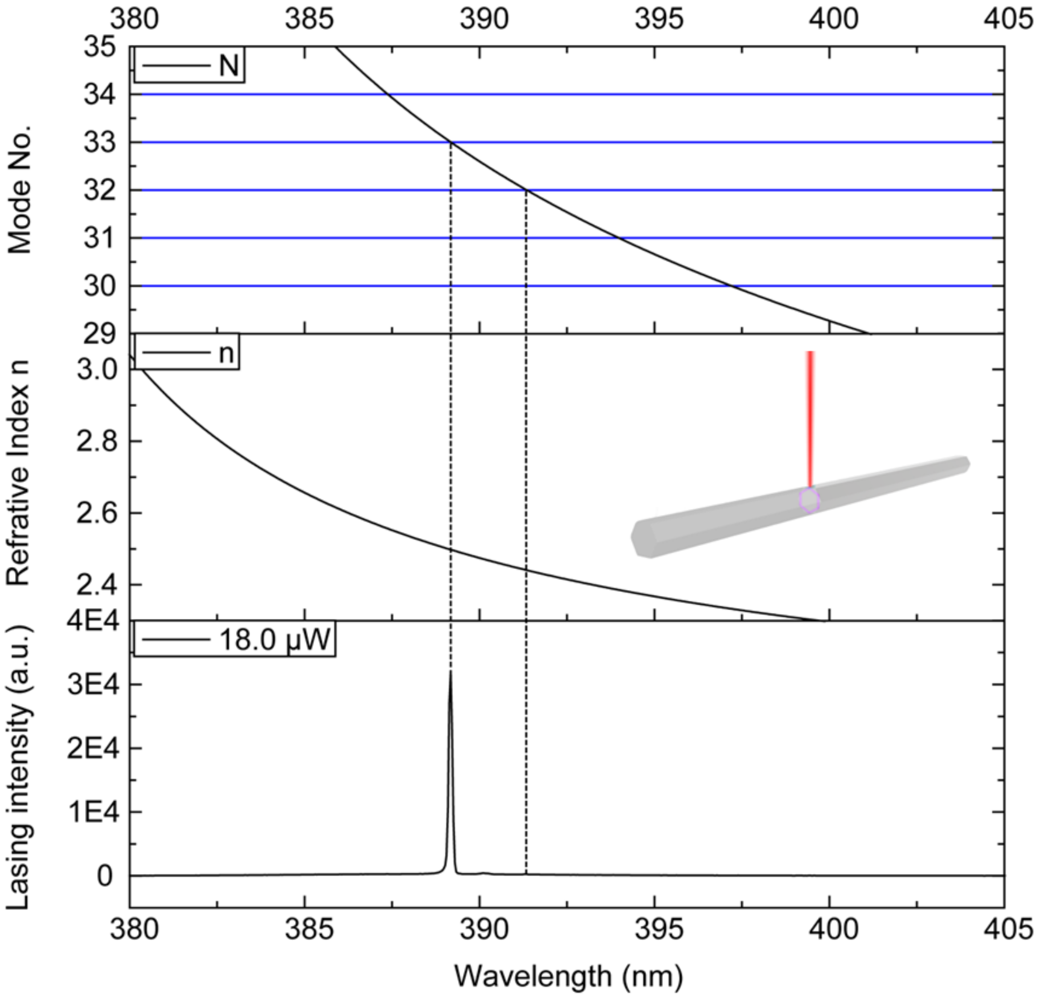

Figure S4. Lasing spectrum (below panel), refractive index (middle panel) and mode number (up panel) as a function of wavelength.

E. Power-dependent lasing spectra under normal and tensile states.

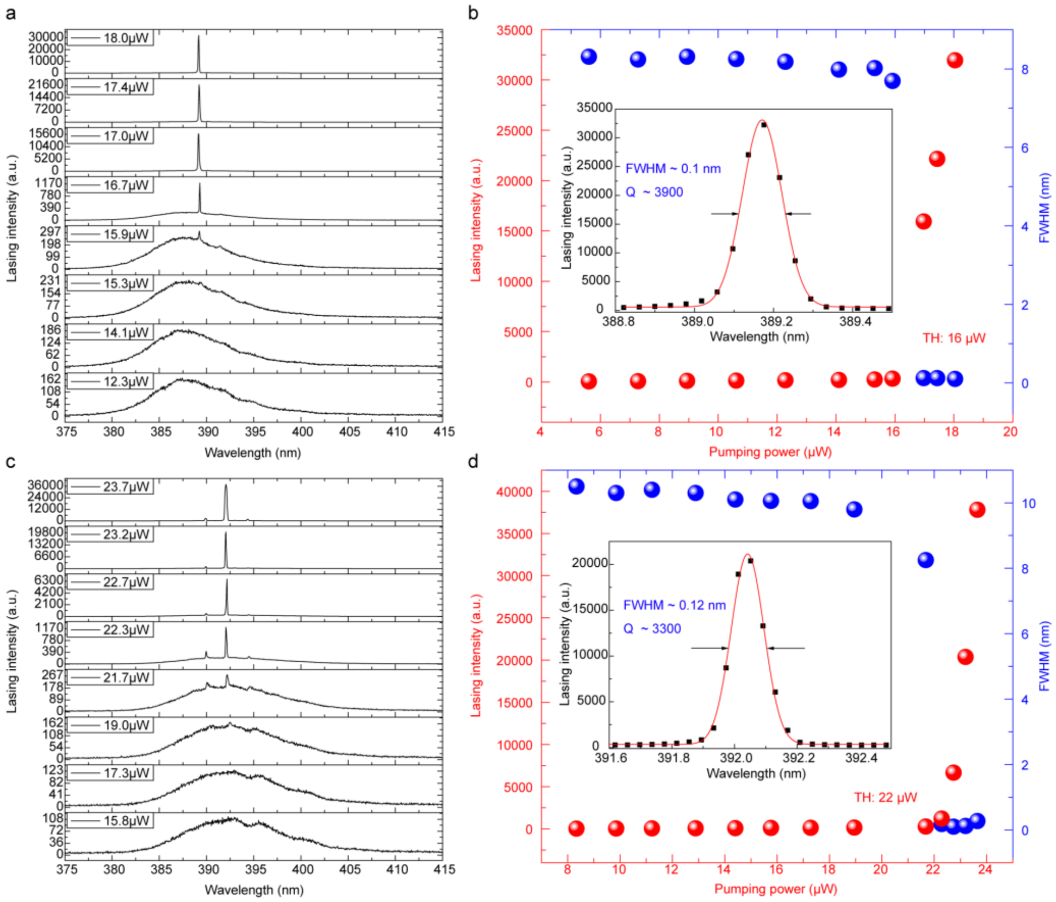

Figure S5. (a, c) Lasing spectra and (b, d) the corresponding input-output characteristics and FWHM as a function of the pumping power for these two cases.

F. Strain-induced refractive index variation and mode shift.

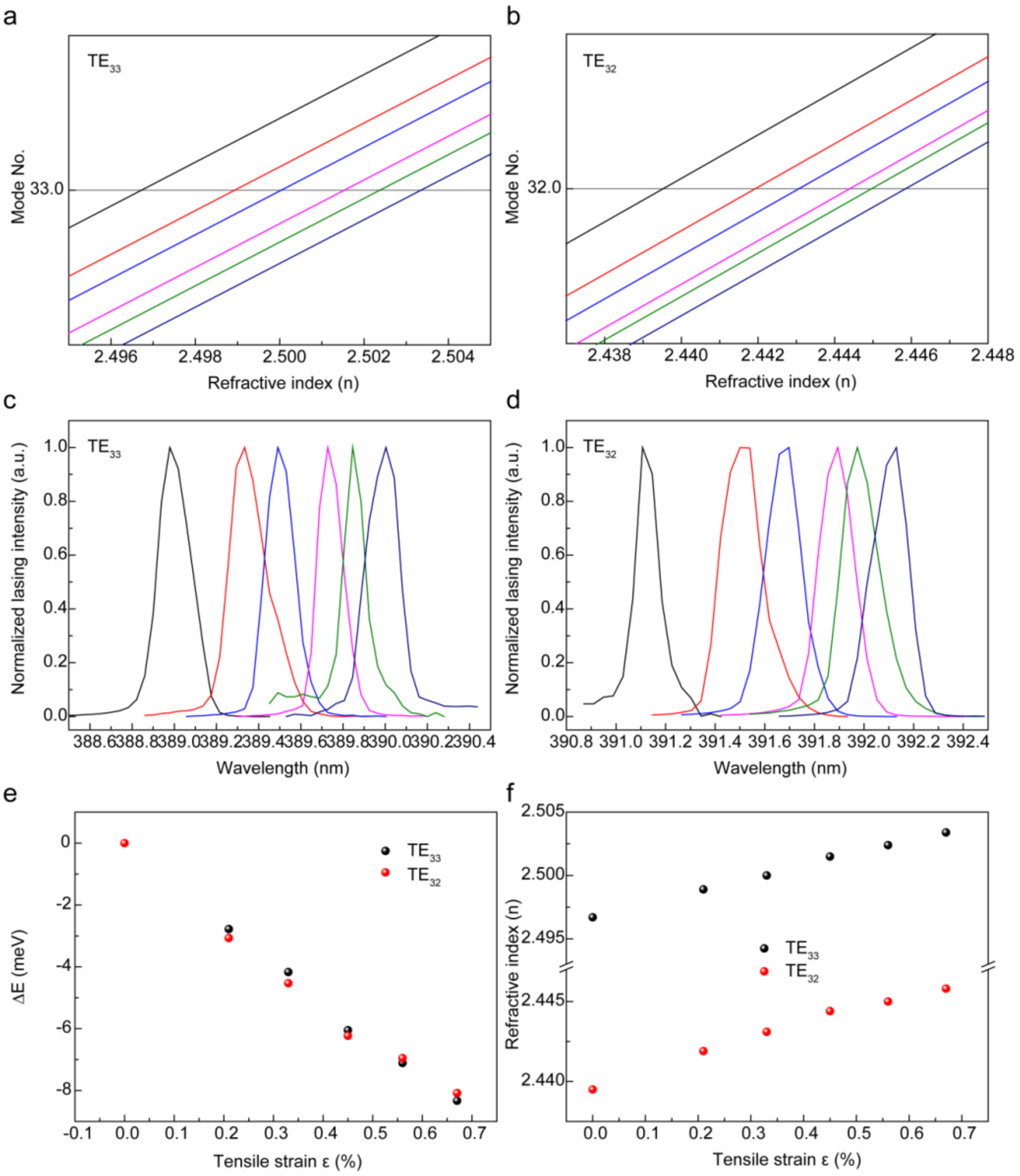

Figure S6. (a, b) the calculated refractive index and (c, d) normalized lasing spectra with increasing of tensile strain for TE_{33} and TE_{32} mode, respectively. (e, f) Dependence of the mode-shift and refractive index on the applied strain for TE_{33} and TE_{32} mode.

G. The calculated mode wavelength with decreasing of diameter.

Table S1. The calculated mode wavelength with decreasing of diameter.

Mode No.	0 %	0.21 %	0.33 %	0.45 %	0.56 %	0.67 %
**33**	388.99	388.94	388.90	388.87	388.85	388.82
**32**	391.12	391.06	391.02	390.98	390.95	390.92

H. Lasing spectra of stress direction perpendicular to *c*-axis of ZnO.

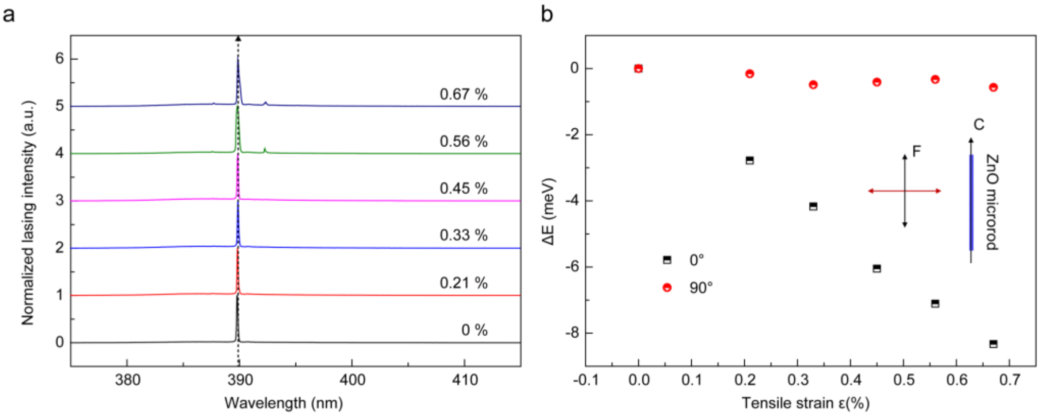

Figure S7. (a) Lasing spectra measured with the included angles of 90° between tensile stress and *c*-axis direction under different tensile strains from 0 % to 0.67 %. (b) The redshift variation for the lasing mode photon energy under two include angles of 0° and 90° as a function of the tensile strain.

I. Power dependent lasing spectra and L-L curve at P⊥C and P∥C state.

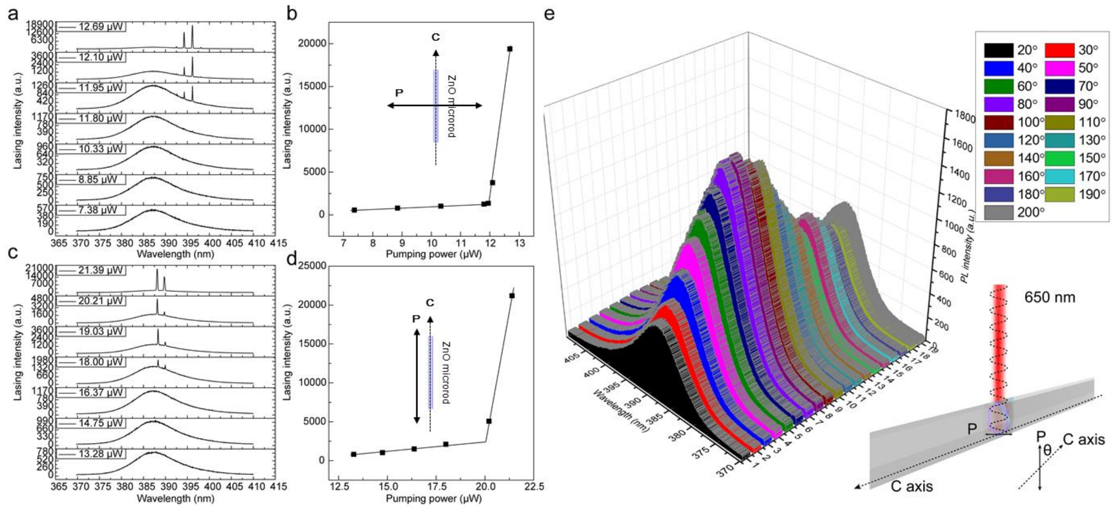

Figure S8. Power dependent lasing spectra and the corresponding input-output characteristics at the state of P⊥C axis (a, b) and P∥C axis (c, d). (e) Polarized-angle dependent PL spectra for single ZnO microcavity.
